# Natural Compounds in Pediatric Disease Treatment

**DOI:** 10.3390/biomedicines14071528

**Published:** 2026-07-08

**Authors:** Dmitry O. Ivanov, Roman O. Shaikenov, Svetlana N. Morozkina, Petr P. Snetkov, Ruslan A. Nasyrov, Polina G. Serbun, Anna D. Kosova, Alexander G. Shavva, Igor M. Kvetnoy

**Affiliations:** 1 Department of Neonatology, Department of Pathology, Saint Petersburg State Pediatric Medical University, Litovskaya Str., 2, 194110 Saint Petersburg, Russia; doivanov@yandex.ru (D.O.I.); rrmd99@mail.ru (R.A.N.); igor.kvetnoy@yandex.ru (I.M.K.); 2 Institute of Advanced Data Transfer Systems, ITMO University, Kronverkskiy Pr., 49, Bldg. A, 197101 Saint Petersburg, Russia; morozkina.svetlana@gmail.com (S.N.M.); ppsnetkov@itmo.ru (P.P.S.); polinaserbunkl@gmail.com (P.G.S.); kosova@itmo.ru (A.D.K.); 3 Progressive Materials and Additive Technologies Center, Kabardino-Balkarian State University Named After H.M. Berbekov, St. Chernyshevsky, 173, 360004 Nalchik, Russia; 4 Institute of Medicine, Saint Petersburg State University, Universitetskaya Emb., 7-9, 199034 Saint Petersburg, Russia; agshavva@yandex.ru; 5 Department of Translational Biomedicine, Saint Petersburg Research Institute of Phthisiopulmonology, Ligovsky Prospect, 2-4, 191036 Saint Petersburg, Russia

**Keywords:** pediatric phytotherapy, medicinal plants, herbal medicine, clinical trials in pediatrics, natural compounds, pediatric therapy

## Abstract

The review evaluates current clinical and epidemiological evidence regarding the use of plant-derived compounds in pediatric practice. Data from randomized controlled trials indicate symptomatic efficacy of selected agents—particularly in acute respiratory infections—alongside generally favorable safety profiles when standardized preparations are used. Emerging research also explores applications in neurodevelopmental disorders, gastrointestinal conditions, and dermatology, and as supportive therapy in pediatric oncology. However, variability in product quality, limited pediatric-specific trials, potential toxicity, and regulatory inconsistencies remain significant challenges. The integration of phytotherapy into pediatric care therefore requires rigorous study design, careful safety monitoring, and clear quality standards to ensure an evidence-based risk–benefit balance.

## 1. Introduction

Natural biologically active compounds derived from medicinal plants are increasingly recognized as pharmacologically active molecules with multitarget mechanisms of action and, in many cases, favorable safety profiles when appropriately used. Their application in pediatric practice has grown steadily over the past decades, reflecting both a global resurgence of interest in phytotherapy and persistent parental demand for “natural” therapeutic options. In the United States, approximately 5% of children use natural products, with the most frequently reported agents including echinacea, cranberry, garlic supplements, and ginseng [1].

Despite widespread use of natural products, important clinical and regulatory challenges remain. Herbal supplements may contain multiple bioactive constituents, and variability in plant species, extraction methods, and manufacturing standards can significantly influence their composition and pharmacological activity [2]. Moreover, although many plant-derived preparations have long histories of traditional use, not all have been rigorously evaluated in well-designed pediatric clinical trials. Some natural products have not demonstrated clear efficacy for specific indications, and certain formulations—such as some teething remedies—have been associated with serious adverse reactions [1]. These concerns underscore the necessity for evidence-based assessment, quality control, and careful risk–benefit evaluation, particularly in vulnerable pediatric populations.

At the same time, a growing body of experimental and clinical research, which will be discussed in this review, supports the role of selected phytotherapeutic agents in managing common pediatric conditions, including respiratory infections, functional gastrointestinal disorders, dermatological diseases, and neurological disorders. In addition, interest is expanding toward the adjunctive use of natural compounds in more complex and severe diseases, including pediatric cancers, where plant-derived molecules may contribute to supportive care, mitigation of treatment-related toxicity, or, in selected contexts, exhibit direct antitumor properties. However, such applications require especially rigorous evaluation due to potential interactions with conventional chemotherapeutic agents and the narrow therapeutic margins characteristic of oncologic treatment [3,4]. Clinical studies currently underway can confirm that the use of herbal remedies for various childhood illnesses, including cancer, is safe when used in reasonable doses and under medical supervision. Examples of such studies will be discussed in Section 5 and Section 6.

In this review, we summarize data from the last two decades regarding natural products used in the treatment of childhood diseases, ranging from common acute conditions to chronic disorders. Particular attention is given to clinical efficacy, safety profiles and restrictions. This review aims to provide a balanced perspective on the rational use of natural compounds in pediatric medicine.

## 2. Methods

This article was conducted as a narrative review of the available evidence on natural compounds in pediatric diseases. To identify the relevant literature, we searched three electronic databases, PubMed, Scopus, and Web of Science, covering the period from January 2000 to December 2025. The search strategy combined terms related to natural products (“natural compounds”, “phytotherapy”, “herbal medicine”, “plant extract”, “flavonoids”) with pediatric terms (“pediatric”, “children”, “infant”, “adolescent”) and specific disease categories (“respiratory infections”, “ADHD”, “atopic dermatitis”, “gastrointestinal diseases”, “oncology”). Additional relevant studies were identified by manually screening the reference lists of retrieved articles and key reviews.

Inclusion criteria were original research (randomized controlled trials, observational studies, case series) or systematic reviews/meta-analyses and studies reporting clinical efficacy, safety, pharmacokinetics, or traditional use of plant-derived compounds. Exclusion criteria were in vitro or animal studies without clinical translation and studies on adult populations only.

## 3. The Use of Plant Substances for the Treatment of Specific Diseases

### 3.1. Respiratory Diseases

Acute infectious diseases are common in childhood and affect various organs and systems. The most common are upper respiratory tract infections, urinary tract infections, and skin infections, necessitating the search for safe and effective treatments [5].

Acute post-viral cough is one of the most common symptoms in childhood and adolescence and frequently causes the seeking of medical help. Cough is a specific protective reflex mechanism of the respiratory tract. Acute cough, whose duration is less than 4 weeks, in most cases is caused by the upper respiratory tract infection (URTI) of viral etiology, which justifies the term “post-viral cough” [6]. Results of pediatric randomized controlled trials support efficiency of honey, a multicomponent product based on *Plantago lanceolata*, *Grindelia robusta*, *Helichrysum italicum* and honey, and *Pelargonium sidoides* in the treatment of acute cough in children [7].

In traditional medicine, according to an ethnobotanical survey in Morocco, plants with a rich phytochemical composition are used to treat coughs: garlic (*Allium sativum*), white wormwood (*Artemisia herba-alba*), European olive (*Olea europaea*), and Moroccan thyme (*Thymus maroccanus*) [8].

Herbal remedies and bee products have shown encouraging results regarding the reduction in the severity and duration of symptoms in respiratory infections in children.

### 3.2. Mental Health and Neurological Disorders

The most common behavioral disorder in children is attention deficit hyperactivity disorder (ADHD), which is characterized by the following main symptoms: hyperactivity, impulsivity, and difficulty concentrating. ADHD is increasingly being diagnosed in children and is becoming a serious problem. The syndrome can also manifest itself in adulthood, significantly affecting the patient’s social life, education, and professional activities. In addition, ADHD is associated with substance abuse, which is why many studies are developing new ways to treat this disorder [9,10].

Among the herbal remedies used for ADHD in children, the most interesting are those that have demonstrated clinically significant effects. French maritime pine bark extract (*Pinus pinaster*) Pycnogenol^®^ improved concentration in children aged 6–14 years after just 1 month of the therapy in a randomized controlled trial, probably due to the modulation of dopamine and norepinephrine. Normalization of antioxidant status and a reduction in oxidative DNA damage were also noted, with minimal side effects [11].

*Bacopa monnieri*, when used for a long time (up to 6 months), led to an observed reduction in inattention and learning problems in most patients in the group, probably because of its neuroprotective and dopaminergic effects. *Ginkgo biloba* and *Valeriana officinalis* showed moderate improvements, but the data from these studies are limited. *Hypericum perforatum* has not demonstrated efficacy in rigorous clinical trials [12,13].

*Panax ginseng* showed significant positive results in randomized studies: inattention and hyperactivity scores decreased, the number of errors in computer testing decreased, and the ratio of theta/beta rhythms in the EEG normalized [14].

A review by Taleb Ali Khalid et al. analyzes the results of a survey of residents of southern Morocco on the use of plants to treat children. It was found that traditional medicine pays attention to the treatment of neurological disorders, among other things. For neurological disorders, including epilepsy and insomnia, the most commonly used plants are *Aloysia citriodora*, *Matricaria chamomilla*, *Papaver rhoeas*, and *Peganum harmala*, accounting for 11.4% of all plants used for these purposes [8].

Based on the comprehensive review by Rigillo et al., the application of natural compounds for the improvement of mental health in children represents a promising frontier in pediatric therapeutics. As the prevalence of anxiety, mood disturbances, and attentional disorders continues to rise among youth, the limitations and safety concerns associated with conventional psychotropic medications have prompted renewed interest in phytotherapeutic alternatives. This review critically covers the evidence from twenty-nine clinical trials investigating natural compounds in pediatric populations, ranging from psychiatric diagnoses such as attention deficit/hyperactivity disorder (ADHD) to subthreshold conditions including nervous agitation, sleep disturbances, and procedural anxiety [15].

The clinical evidence, while heterogeneous in quality, identifies several medicinal plants with therapeutic potential. For ADHD management, *Crocus sativus* (saffron) demonstrated efficacy comparable to methylphenidate in multiple randomized controlled trials [16], while *Pinus pinaster* (Pycnogenol^®^) showed improvements in attention and hyperactivity alongside favorable effects on oxidative stress markers [11,17]. *Ginkgo biloba* and *Bacopa monnieri* exhibited nootropic properties with preliminary benefits for cognitive symptoms, though results remain inconclusive [18,19]. For subthreshold symptoms such as restlessness and dyssomnia, fixed combinations of *Valeriana officinalis* and *Melissa officinalis* significantly improved sleep quality and reduced agitation in large observational studies [20]. Notably, inhalation of *Lavandula angustifolia* essential oil consistently reduced the procedural anxiety and pain perception in several RCTs, with measurable effects on cortisol levels and vital signs [15,21].

Complementing these clinical findings, bioinformatic analyses revealed that these botanicals exert their effects through complex multipharmacological mechanisms. Rather than acting on single molecular targets, phytocompounds modulate multiple pathways simultaneously, including neuroinflammatory cascades (NF-κB, matrix metalloproteinases), neurotransmitter systems (MAO-A, GABA receptors), and neuroprotective signaling (BDNF, ERK pathways). This multitarget activity may explain their favorable safety profiles and potential utility in addressing the heterogeneous symptomatology of pediatric mental health disorders [15].

The current evidence supports a role for standardized herbal preparations as complementary tools in child and adolescent mental health care, particularly for the mild-to-moderate symptoms where conventional pharmacotherapy may be ineffective. However, significant gaps remain regarding the optimal dosing, long-term safety, and pharmacokinetics.

### 3.3. Gastrointestinal Diseases and Disorders

The use of natural compounds, particularly herbal products, for the management of common childhood conditions such as infant colic and flatulence represents a longstanding tradition in pediatric home care. A recent comprehensive questionnaire-based study combined with chemical analysis by Bulut and colleagues provides critical insights into both the prevalence of this practice and the potential safety concerns associated with it, specifically regarding pyrrolizidine alkaloid (PA) contamination [22].

**Figure 1:**
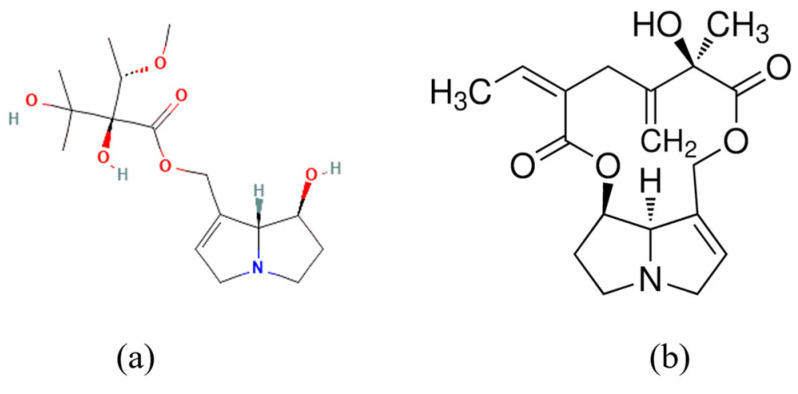
Chemical structure of (**a**) europine and (**b**) seneciphylline.

The study, conducted at Gazi University Hospital Pediatrics Clinics, surveyed 124 parents of infants aged 0–1 years to evaluate the frequency and patterns of herbal product use for gas pain. The results revealed that 31.5% of parents administered herbal products to their children for this purpose, with *Foeniculum vulgare* (fennel) being the most preferred plant (51.3%), followed by mixtures containing cumin and dill. This preference aligns with the fennel’s documented carminative properties and its traditional use in pediatric gastrointestinal complaints [23]. Notably, the majority of parents (59%) procured these products from spice shops rather than pharmacies, and 97.4% administered them internally. While parents cited the perceived usefulness and the “natural and harmless” nature of these products as primary reasons for their choice, a disturbing finding was that a significant portion relied on doctors’ recommendations, suggesting that medical professionals may inadvertently recommend products of questionable quality [22]. The analytical component of the study evaluated 28 herbal products commonly used for infant gas pain, purchased from spice shops, markets, and online sources. Using LC-QTOF-MS analysis, researchers detected the presence of pyrrolizidine alkaloids—specifically europine and seneciphylline (Figure 1)—above the quantifiable limit of 10 μg/kg in 75% of samples. Particularly alarming was the detection of europine in all fennel samples analyzed, with concentrations reaching 328.235 μg/kg in a cumin sample and seneciphylline levels up to 434.067 μg/kg in a chamomile sample. When evaluated against the German Federal Institute for Risk Assessment’s recommended maximum daily intake of 0.007 μg/kg body weight, these contamination levels pose significant risks [24]. For a 5 kg infant, consumption of just 2 g of the most contaminated tea would result in pyrrolizidine alkaloid (PA) exposure approximately 25 times above the safe limit, with cumulative effects potentially occurring over extended periods of use.

This contamination likely stems from the inadvertent co-harvesting of PA-producing plants from families such as Boraginaceae, Asteraceae, and Fabaceae, which grow alongside medicinal crops. The findings underscore a critical disconnect between traditional usage patterns and modern safety standards, demonstrating that “natural” products are not inherently safe for vulnerable pediatric populations. Given that PAs are known to cause hepatotoxicity, developmental toxicity, and genotoxicity, the long-term consumption of contaminated herbal products by infants represents an underrecognized public health concern [22].

Taleb Ali Khalid obtained similar data in Morocco. In the treatment of digestive disorders in children, the most frequently used herbs are chamomile (*Matricaria chamomilla*) (22.7%), fennel (*Foeniculum vulgare*) and anise (*Pimpinella anisum*) both 18.2% [8].

### 3.4. Use in Chemotherapy

Although chemotherapy is prescribed according to the severity of the disease and the individual characteristics of the patient, side effects often occur during the treatment. Anticancer drugs are often non-selective, leading to systematic side effects, especially in children, who experience renal dysfunction caused by drugs such as cisplatin, ifosfamide, carboplatin, and methotrexate. The mechanisms of renal impairment include a variety of processes: vascular damage, obstruction, and renal tubular dysfunction [25].

Many plant compounds have pronounced antioxidant properties and are capable of neutralizing the active forms of oxygen that are formed by chemotherapeutic drugs or their metabolites [26]. In this regard, plant compounds are being actively studied as agents for the reduction of the side effects of chemotherapy. Saffron extract has been shown to reduce cardiomyocyte apoptosis when exposed to doxorubicin. Traditional plant formulas reduce intestinal inflammation caused by irinotecan by regulating signaling pathways, strengthening intercellular contacts, and reducing the production of pro-inflammatory cytokines. Black rice anthocyanins, EGCG, and other phytochemicals demonstrate a cardioprotective effect, including through activation of the Nrf2/ARE pathway, as was described in the review by Lin et al. [27].

**Figure 2:**
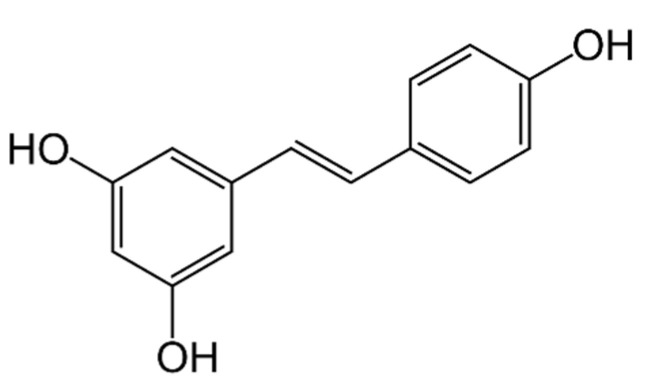
Chemical structure of resveratrol.

A number of plant substances reduce the nephrotoxicity of cisplatin. Resveratrol (Figure 2) and catechins reduce DNA damage and apoptosis of hematopoietic cells [28]. Conifer extracts and phenolic compounds reduce photosensitivity and radiation damage [29]. Thus, phytocomponents can probably improve the tolerability of anticancer therapy and potentially allow reducing its dose, which is especially important in pediatrics.

However, some plant compounds may be more toxic to children than to adults, especially in terms of hepatotoxicity. For example, there have been cases of children being poisoned by essential oils when drinking tea whose composition had not been properly checked [30]. Therefore, combinations of chemotherapy drugs and plant components require the extensive safety research.

Also, there is a tremendous need in clinics to impair cancer progression through noninvasive therapeutic approaches. The use of natural compounds to achieve this is of importance to improve the quality of life of young patients during the treatments. Ferrucci et al. analyzed the molecular targets and signaling pathways, and proposed the mechanisms of action of phytochemicals that have shown promising results in preclinical and selected clinical studies. Among the most studied substances are chebulagic acid, apigenin, norcantharidin, crocin (saffron), parthenolide, longikaurin E, lupeol, spongistatin 1, and deoxy-variolin B. In addition, the authors draw attention to the potential of nutraceutical compounds, including agaritin, Ganoderma components, diallyl trisulfide and ajoene from garlic, epigallocatechin gallate from green tea, curcumin, resveratrol, and quercetin—as possible adjuvants to the anticancer therapy [31].

### 3.5. Dermatological Disorders

Skin diseases such as cancer, dermatitis, psoriasis, wounds, acne, and skin infections are widespread and have significant social and economic consequences. Therefore, the development of plant-based drugs against this broad group of diseases is a promising area of research [32].

Atopic dermatitis is a serious public health problem, causing significant physical and psychological impact on patients. Natural compounds have traditionally been widely used to treat a variety of conditions, including atopic dermatitis.

**Figure 3:**
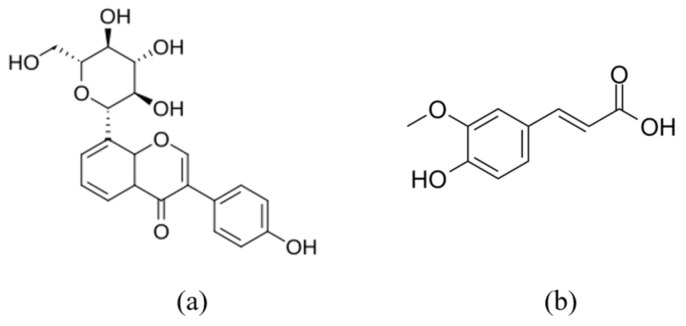
Chemical structure of (**a**) puerarin and (**b**) ferulic acid.

A literature review demonstrates that many natural compounds, particularly puerarin [33], ferulic acid [34] (Figure 3) and ginsenosides [35], have a protective effect against atopic dermatitis, as supported by the preclinical studies [36].

The survey of Morocco residents revealed that for the treatment of dermatological diseases, including eczema and burns, the most frequently prescribed agents are onion (*Allium cepa*), henna (*Lawsonia inermis*) and olive (*Olea europaea*), accounting for 14.6% of all agents used [8].

## 4. Use of Herbal Ingredients in Pediatrics in Different European Countries

The use of herbal remedies in pediatrics varies depending on the cultural context, level of medical integration, and characteristics of the healthcare system. In some regions, herbal medicine retains its character as an ethnomedical tradition, while in other countries it is integrated into the formalized medical practice. Taleb Ali Khalid’s review provides extensive information on the use of plants in southern Morocco [8]. There are also other reviews that examine an area within a single country.

### 4.1. Romania

Ethnopediatrics represents a crucial intersection of cultural heritage and biomedical potential, yet systematic investigations remain scarce, particularly in Eastern Europe. A recent study by Petran et al. provides quantitative insights into the contemporary use of medicinal plants for children’s diseases in Southern Romania, a region with rich ethnomedical history. Through structured interviews with 326 mothers of hospitalized children, the research documents current practices and analyzes the sociodemographic factors influencing them.

The study identified 25 medicinal plant species from 15 families currently employed in pediatric care. The Use-value citation index (UVc) evaluates the relative importance of each species based on its cited uses. The most frequently cited species were *Mentha* spp. (UVc = 0.509), *Matricaria* spp. (UVc = 0.301), and *Calendula officinalis* (UVc = 0.365), used primarily for digestive, respiratory, and dermatological conditions. Quantitative ethnobotanical indices confirmed their cultural significance; Informant Consensus Factor was exceptionally high for skin disorders (0.99) and digestive diseases (0.98), while Fidelity Level analysis revealed highly specific applications: *Mentha* spp. for diarrhea (93.3%), *Matricaria* spp. for infantile colic (68%), and *C. officinalis* for diaper dermatitis (89.1%) [37].

The treatment of functional gastrointestinal disorders, particularly infantile colic, represents a well-preserved domain of ethnopediatric knowledge. Mothers reported administering *Matricaria* spp. as early as 1.5 months of age, often in polyherbal combinations with *Foeniculum vulgare*, *Carum carvi*, and *Anethum graveolens* seeds. This empirical combination of carminative plants finds partial support in clinical evidence: a single randomized trial suggested the effectiveness of *F. vulgare* combined with *Matricaria recutita* and *Melissa officinalis* for infant colic, though confirmatory studies are lacking. *Mentha* spp. leaves combined with chamomile were employed for diarrhea from three months of age.

For respiratory diseases, *Tilia tomentosa* flowers were utilized for cough (FL = 92.5%), while *Calendula officinalis* ointment applied from the first month of life for diaper dermatitis has been examined in randomized trials that reported some advantages over aloe, although the evidence is limited. *Arnica montana* was used exclusively for traumatic injuries (FL = 100%), with some reports suggesting the efficacy in pediatric soft-tissue bruising, though robust clinical trials are lacking.

Comparison with historical data reveals dramatic knowledge contraction, from 153 documented species in Romanian ethnopediatrics (1860s–1970s) to only 25 currently in use, reflecting urbanization, reduced oral transmission, and displacement by conventional medicines. Nevertheless, 24 of 25 reported use cases are consistent with available scientific evidence, and 88% of respondents reported improvement after herbal therapies.

Multivariate analysis identified maternal education as the sole independent determinant of both the number of plants employed and the variety of ailments treated. Higher-educated mothers used greater plant diversity and were more likely to harvest personally, challenging assumptions about traditional knowledge being confined to less-educated populations. Rural background predicted higher overall use but not greater therapeutic diversity. Of the 25 documented plants seven were administered to infants under three months, underscoring safety concerns. While *F. vulgare*, *Matricaria* spp., and *C. officinalis* are generally recognized as safe, others warrant caution: excessive maternal *P. anisum* use has been associated with neonatal toxicity, and *A. ursinum* carries gastrointestinal toxicity risks. Clinical pediatric studies are lacking for 21 of the 25 species. Thus, medicinal plants continue to play a meaningful role in the treatment of childhood diseases in Southern Romania [37].

### 4.2. Germany

Epidemiological studies confirm that the use of herbal medicines among children occupies a significant, albeit relatively limited, place in pediatric practice in Germany. For example, in the KiGGS study, which included 17,450 participants aged 0 to 17 years, 5.8% of children took at least one herbal medicine during the previous week. The frequency of use was highest among children under 6 years of age, reflecting the traditional perception of herbal medicines as “mild” and safe [38].

Most of the herbal remedies used are aimed at the symptomatic treatment of respiratory diseases. About two-thirds of all registered preparations were used for coughs, colds, and acute upper respiratory infections, while other indications occurred in less than 5% of cases. Almost half of the preparations were prescribed by doctors, which emphasizes their integration into official medical practice, while the rest were used independently or on the recommendation of other specialists. Side effects were extremely rare: only 0.9% of uses were accompanied by adverse reactions, which is comparable to the frequency of side effects in conventional medicines.

Thus, the epidemiological data demonstrate that herbal preparations in pediatrics are used primarily for the treatment of mild and often self-limiting diseases, with a relatively low risk of side effects, especially in younger children. It is important to take this information into account when interpreting the results of clinical studies aimed at evaluating the effectiveness of specific phytochemicals, as well as when formulating recommendations for the safe use of herbal remedies in pediatric practice. At present, herbal preparations in Germany are used as symptomatic medications for mild illnesses [38].

### 4.3. United Kingdom

Systematic epidemiological evidence confirms that the use of complementary and alternative medicine (CAM), including herbal products, is highly prevalent among pediatric patients in the United Kingdom. A comprehensive systematic review by Posadzki et al. analyzed 11 surveys published between 2000 and 2011, encompassing a total of 17,631 pediatric patients. The methodological quality of the included surveys was generally poor, and significant heterogeneity precluded formal meta-analysis. Nevertheless, across surveys on CAM in general, the average one-year prevalence rate was 34% (range 20–41%), while the average lifetime prevalence reached 42% (range 29–61%). Notably, studies with sample sizes exceeding 500 participants reported considerably lower prevalence rates (one-year 30.5%, lifetime 29%) compared to smaller studies (41% and 49%, respectively), suggesting that larger, more representative samples yield more conservative estimates [39].

Across all surveys, herbal medicine was identified as the most popular CAM modality. Using a ranking method that averaged modality popularity across studies, herbal medicine ranked first in 36.3% of surveys, with an average reported use of 21.3%. The specific herbal medicines most frequently administered to children included *Echinacea* spp., *Aloe vera*, *Oenothera biennis* oil, Chinese herbal mixtures, *Linum usitatissimum* oil, *Borago officinalis* seed oil, *Dolichandra unguis-cati*, grapeseed/pinebark extract, cayenne capsules, colostrum, herbal teas, *Glycyrrhiza glabra* root, and lobelia. These were employed primarily for respiratory infections, inflammatory bowel disease, atopic dermatitis, and general pediatric ailments [39].

Perceived effectiveness of CAM was reported in seven surveys (63.6%), with an average of 48.3% (range 14–61%) of parents or patients stating that CAM was beneficial. However, the review authors note that critical evaluation of the evidence does not suggest any of the commonly used herbal medicines are remarkably effective for pediatric indications. Adverse effects associated with CAM use were documented in only two surveys (18.1%), with an incidence of 17.5% (range 5–30%). This figure likely underestimates true risk due to underreporting and the lack of systematic safety monitoring in most studies. Costs of CAM were reported in three surveys, but variability in the reporting prevented meaningful aggregation [39].

Predictors of CAM use were examined in seven surveys. Consistent with findings from Southern Romania, higher maternal or parental education emerged as a significant predictor of CAM use in the majority of these studies. Additionally, friends and family were the most common source of advice about CAM (reported in five out of nine surveys), followed by healthcare professionals and media. Importantly, several surveys found that a substantial proportion of parents (ranging from 54% to 66%) did not disclose their child’s CAM use to medical practitioners, highlighting communication gaps that could lead to potential herb–drug interactions or delayed diagnosis. In one large longitudinal cohort of 13,988 children (the ALSPAC study), 11.8% of children received homeopathic products by age 8.5 years, with Chamomilla (31.8%) and Arnica (32.2%) being the most frequently used, underscoring the overlap between herbal and homeopathic preparations in the UK context [39].

The review concludes that while there is a paucity of high-quality surveys, the available data indicate that CAM use—particularly herbal medicine—is considerable among UK pediatric patients. Pediatricians are advised to acquire sufficient knowledge in this area to advise parents responsibly. The authors call for future surveys employing uniform definitions of CAM, validated outcome measures, random sampling, and representative samples to generate more reliable prevalence estimates. Thus, in the United Kingdom, herbal medicinal products are widely used by children, primarily for self-limiting conditions, but the evidence base for their efficacy remains limited, and safety concerns persist [39].

### 4.4. Italy

Ethnobotanical knowledge in central and southern Italy remains highly conservative, particularly in rural areas. A systematic review by Motti et al. analyzed 34 studies published between 1978 and 2017, documenting the use of medicinal plants specifically for pediatric healthcare. In total, 83 taxa from 37 families were recorded across 116 use reports. According to ISTAT data (2005), 10% of Italian children aged 0–14 years received some form of complementary medicine, with 2% using herbal remedies directly [40].

Geographically, Tuscany exhibited the highest number of plant use reports and the greatest species diversity, followed by Campania and Sicily. *Allium sativum* (garlic) was the most widely cited species, reported in eight regions. Other frequently used plants included *Malva sylvestris* (six regions), *Matricaria chamomilla* (six regions), *Juglans regia* (five regions), and *Ruta* spp. (five regions). Eight ailment categories were identified, with gastrointestinal complaints dominating (47.8% of all reports), followed by skin disorders and anthelmintic applications. Notably, anthelmintic uses comprised 45% of all medicinal treatments, reflecting the persistent prevalence of helminth infections in rural areas of southern Italy, particularly Sicily [40].

The most frequently used plant parts were leaves and fruits (19.5% each), followed by aerial parts (16.9%) and flowers (13.6%). Raw consumption (42%) and decoction (33%) were the predominant preparation methods, while oral administration (56%) and topical application (37%) were the main routes. For gastrointestinal ailments in children, *Matricaria chamomilla* and *Malva sylvestris* were employed as anti-inflammatory and soothing agents. For skin conditions, particularly diaper dermatitis and cradle cap, *Juglans regia* leaves and *Myrtus communis* essential oil were applied topically. *Allium sativum* was administered raw or as a decoction for helminthiasis, a practice consistent with its documented polyphenol-rich anthelmintic activity. Teething pain was managed by rubbing *Rosa canina* juice on gums or using *Althaea officinalis* roots as pacifiers, though mechanical rather than anti-inflammatory effects are likely for the latter [40].

Several traditional uses lack robust scientific validation. For instance, *Cupressus sempervirens* and *Juniperus communis* are added to bath water to strengthen children’s legs and promote first steps—a practice with no clinical evidence. Similarly, *Nasturtium officinale* is used for pediatric anemia without proven efficacy. The review highlights that while some herbal remedies are administered to both adults and children at reduced dosages, others may be potentially harmful from a modern clinical perspective. Nevertheless, the ethnobotanical heritage of central and southern Italy continues to inform pediatric self-care, particularly in rural communities, and warrants further pharmacological investigation [40].

## 5. Regulation and Safety of the Use of Herbal Ingredients in Pediatrics

Herbal compounds are not as potent as conventional ones, which makes them suitable for the use in treating children, but incorrect or excessive use can lead to toxic effects even in the case of herbal preparations. Therefore, the use of herbal compounds in pediatrics should be regulated on the basis of clinical studies and trials.

The issue of the safety of herbal medicines in pediatric practice requires a balanced and scientifically sound approach. Most medicinal plants do not have pronounced toxicity when standard dosages and correct preparation techniques are followed. However, violations of drug preparation methods can lead to the formation of potentially toxic compounds. Particular caution should be exercised in the use of plants such as *Peganum harmala* and *Nerium oleander*, which pose an increased risk to children. Traditional knowledge of folk medicine, including accumulated empirical experience, is valuable as a source of information, but its integration into the formal healthcare system is difficult due to the lack of standardization and formal regulation [8].

There are well-known data about natural compounds that have been defined as endocrine-disrupting chemicals (EDCs) due to their hormonal activity and influence on the endocrine system. Their “hormone-like action” may act on molecular, cellular, and epigenetic functions of the endocrine system. Even seemingly safe natural products can have hormonal activity and cause rather severe endocrine disorders such as premature breast development and gynecomastia.

This was the reason for establishing guidelines for EDCs regarding daily intake in food. The rate of EDC-associated diseases has increased over the last few decades. Children may receive EDCs via the transplacental route from their mother or from milk [41]. Beyond synthetic chemicals, certain natural compounds themselves can act as endocrine disruptors, interfering with hormone synthesis, transport, or receptor signaling. EDCs bind to nuclear hormone receptors (estrogen, androgen, thyroid hormone receptors), leading to altered gene transcription, epigenetic changes, and disrupted hormonal balance. The developing organism is especially vulnerable: in utero, early postnatal life, and puberty are critical windows during which EDC exposure can permanently reprogram endocrine and metabolic set points, contributing to later health issues such as altered growth, precocious or delayed puberty, thyroid dysfunction, obesity, and metabolic syndrome [41]. Therefore, even products derived from plants—if contaminated, improperly prepared, or used at high doses—may pose endocrine risks to children, underscoring the need for rigorous safety evaluation.

According to preclinical and clinical studies, which will be discussed in this section and Section 6, many natural compounds demonstrate high safety. Randomized controlled trials and prospective observations confirm the good tolerability of standardized herbal preparations in pediatrics. A narrative review by Murgia et al. [7], which pooled data from multiple studies involving more than 18,000 patients (60.3% children), reported no serious adverse events with Asteraceae-based preparations. Similarly, *Echinacea purpurea* preparations showed good tolerability without serious side effects, and bee products in clinical studies were characterized by rare and mild adverse reactions [5].

At the same time, certain substances, including puerarin and pseudoephedrine, may cause side effects and require careful monitoring, especially in children. Despite the promising potential of a number of compounds (baicalein, quercetin, piperine, indirubin, ginsenosides, etc.), their widespread introduction into clinical practice is only possible with additional high-quality research. This is especially true for chronic dermatological diseases, where the need for safer therapeutic strategies remains high [36].

Age restrictions should also be taken into account. Honey is contraindicated for children under one year of age due to the risk of infant botulism. When applied in high concentrations, mint and eucalyptus essential oils can cause laryngospasm and bronchospasm, central nervous system depression, and respiratory failure in infants [7].

Regulatory aspects of the use of natural remedies remain a complex and ambiguous area. The general system of regulation of chemical substances, including components of natural origin, is characterized by fragmentation and insufficient effectiveness [41]. The lack of strict registration requirements for many cold and cough remedies contributes to the emergence of products with insufficiently studied quality and safety. An additional complication is the vagueness of the concept of “natural,” which makes it difficult to objectively evaluate a product based on its labeling [7].

Given the limited evidence base, the monographs of the European Scientific Cooperative on Phytotherapy (ESCOP), which summarize data on the long-term safe use of medicinal plants, are of particular importance. When selecting preparations for children, it is important to ensure that the ethanol content complies with the recommendations of the European Medicines Agency (EMA), that there are no synthetic preservatives, and that standardized plant raw materials are used [7].

### Study of the Interaction of Drugs and Herbal Substances

Studies on the efficacy of medicinal components when used in combination with chemical drugs and medical techniques are of particular interest. Such research not only helps to understand the safety of potential interactions between the two components but also identifies possible synergistic or, conversely, antagonistic effects (Table 1).

One of the key aspects of the safety of herbal preparations in pediatric dialysis patients is their potential interaction with anticoagulants, as well as their effect on the hemostatic system and the metabolism of concomitant medications. A study involving 28 children with end-stage chronic kidney disease aged 10–18 years undergoing regular hemodialysis evaluated the effect of curcumin at the dose of 1 g/day for 3 months on inflammatory and oxidative biomarkers, as well as on indicators of liver, kidney, and coagulation system function. In this clinical trial, no statistically significant differences were found between the curcumin and placebo groups regarding the effect on blood coagulation after 3 months of the therapy. All coagulation parameters remained within normal ranges and were comparable in both groups [42].

Another combined study included 85 children (ages 7–15) with persistent bronchial asthma. Patients received either the herbal formula CUF2 (an extract of five herbs: *Astragalus mongholicus*, *Cordyceps sinensis*, *Radix stemonae*, *Bulbus frutillariae cirrhosae*, *Radix scutellariae*) in capsules or a placebo for 6 months alongside baseline therapy with inhaled corticosteroids. After 6 months, no statistically significant differences were found between the groups in terms of the reduction in the dose of inhaled steroids, changes in the severity scale, or FEV1/FVC (Forced expiratory volume/Forced vital capacity) and peak expiratory flow rate. Biochemical markers (eosinophils, total IgE, house dust mite-specific IgE, IL-18, TARC—thymus and activation-regulated chemokine) also did not differ between the groups. Thus, the addition of CUF2 to inhaled steroids did not provide any additional clinical benefit. Transient complaints of dry mouth and throat were reported in 39 of 42 patients in the CUF2 group and in 33 of 43 in the placebo group; episodes of nosebleeds were observed in five and 11 children, respectively, with no differences in the laboratory parameters (complete blood count, liver and kidney function tests). It is important to emphasize that concomitant use of inhaled corticosteroids did not lead to additional adverse events when the herbal preparation was added, and no clinically significant drug interactions were noted that could have exacerbated steroid-induced side effects [43].

Some studies of plant substances focus on the components of traditional Chinese medicine. A systematic review showed that the addition of herbal therapy to the standard epilepsy treatment in children (valproate, carbamazepine, oxcarbazepine) significantly increases overall efficacy compared to drug monotherapy. At the same time, the incidence of adverse events in the combination therapy group was significantly lower than in the drug-only group, based on four studies (n = 246). Other studies confirmed a reduction in adverse events with the addition of herbal therapy. An analysis of reported adverse events in 10 studies identified the following effects in the combination therapy group (herbal therapy + pharmaceuticals): dyspepsia, nausea, vomiting, drowsiness, dizziness, memory impairment, altered consciousness, lethargy, and elevated liver transaminases; in the drug monotherapy group, headaches and weight gain were additionally noted. It is important to emphasize that no serious adverse events were reported in the studies using only herbal preparations. However, the review authors point out the high risk of systematic error: experimental data require further verification and validation [44].

Another study examined the efficacy of Chinese herbal medicines in oncology. A review by Ximeng Li et al., which included 49 randomized controlled trials, evaluated the addition of Chinese herbal medicine to standard anticancer therapy in children with hematologic and solid malignancies. The combination with herbal preparations significantly increased the rate of complete and partial remissions compared to chemotherapy alone. The most pronounced effect was observed for immunostimulatory herbs and tonic mixtures. Phytotherapy improved immune parameters: CD4+ levels and the CD4+/CD8+ ratio increased. A significant increase in platelets and neutrophils was also observed, indicating a reduction in myelosuppression. A key finding was a statistically significant reduction in the incidence of infections, nausea/vomiting, and ECG rhythm abnormalities in the phytotherapy group. The drug Kangfuxin (*Periplaneta americana* extract) was particularly effective in the acceleration of the healing of oral ulcers. Despite the absence of a reported increase in adverse events, the long-term safety of combining herbal medicine with chemotherapy in children remains incompletely studied, as there was a lack of the standardization of formulations in the studies [45].

Selected studies are gathering important information on contraindications of herbal components during chemotherapy. A review by Ayush Gandhi et al., summarizing clinical and preclinical data on interactions between natural products and chemotherapy, targeted drugs, and immunotherapy, emphasizes that the most dangerous interactions occur through the induction or inhibition of cytochrome P450 (especially CYP3A4) and P-glycoprotein. St. John’s wort (*Hypericum perforatum*), which contains hyperforin, acts as a potent inducer of CYP3A4 and P-gp, making it absolutely contraindicated during anticancer therapy. Grapefruit juice, on the other hand, inhibits intestinal CYP3A4, increasing the bioavailability of nilotinib and sirolimus with a risk of myelosuppression. Pharmacodynamic interactions present a separate challenge: high doses of green tea (EGCG) directly bind to bortezomib, inactivating its function. Curcumin shows conflicting data—ranging from the increased cytotoxicity of 5-fluorouracil to possible neutralization of reactive oxygen species necessary for certain chemotherapeutic agents. Ginseng (*Panax ginseng*) inhibits several isoforms of cytochrome P450 (CYP) and, in clinical cases, is associated with hepatotoxicity when taken concurrently with imatinib, while garlic in high doses enhances the antiplatelet effect, increasing the risk of bleeding in the context of thrombocytopenia [46].

The authors propose the classification of natural products into three categories. St. John’s wort, grapefruit juice, and high-dose green tea extracts are classified as high-risk; their concurrent use with chemotherapy or targeted therapies must be strictly avoided. Moderate risk (ginseng, curcumin, echinacea) is characterized by mixed or limited clinical data, requiring cautious use and monitoring of liver function, coagulation, and glucose levels. Low risk (ginger in culinary doses) is considered safe for relieving nausea, although high doses require caution when taken concurrently with anticoagulants. A key problem remains patients’ failure to disclose their use of dietary supplements—more than half of cancer patients do not inform their doctor about their use, which creates a risk of unexplained adverse events or reduced treatment efficacy [46].

However, in some cases, herbal substances help manage the severe negative effects of chemotherapy. In a double-blind, randomized, placebo-controlled trial, 141 children aged 5–15 years with newly diagnosed acute lymphoblastic leukemia (ALL) received either curcumin at the dose of 3 mg/kg twice daily (n = 71) or a placebo (n = 70) for 3 months alongside standard chemotherapy with vincristine (1.5 mg/m^2^ weekly). In the curcumin group, neuropathy developed in 39.4% of patients, whereas in the placebo group, it occurred in 70.0% (*p* < 0.001). Analysis of nerve conduction showed that the frequency of motor disturbances was significantly lower in the curcumin group (30/284 affected nerves versus 54/280 in the placebo group, *p* = 0.012). Electromyographic signs of lower limb myopathy were observed in 8.5% of patients in the curcumin group versus 28.6% in the placebo group (*p* = 0.002) [47].

Curcumin proved safe for pediatric patients: the incidence of gastrointestinal complications (diarrhea, anorexia, constipation, vomiting) did not differ between groups (*p* > 0.05), and mild transient symptoms resolved without intervention. No patient was excluded due to serious adverse events. The authors suggest that the neuroprotective effect of curcumin is mediated by its antioxidant activity, as well as by the inhibition of TNF-α and nitric oxide, which has been confirmed in animal models of vincristine-induced neuropathy. Unlike previous review studies, in which curcumin was considered a potential inhibitor of CYP3A4, this study found no clinically significant pharmacokinetic interaction with vincristine: side effects were not exacerbated, and antitumor efficacy was not reduced [47].

Thus, phytotherapy in pediatrics is generally characterized as an effective method. However, the need for strict quality control, consideration of age restrictions, and further clinical research remains fundamentally important for minimizing risks and ensuring the rational use of herbal remedies in children.

## 6. Clinical Studies on the Efficacy of Herbal Components in Pediatrics

Data from randomized controlled trials confirm the clinical potential of certain herbal preparations in acute respiratory infections and other diseases in children (Table 2). According to the systematic reviews, the use of honey in children older than one year effectively reduces the severity and duration of nighttime cough associated with upper respiratory tract infections and improves sleep quality in both children and their parents. Honey has been shown to be superior to no treatment and diphenhydramine; comparative studies have also noted its advantage over dextromethorphan [5,7]. Additional randomized studies have demonstrated that medical devices based on polysaccharides, resins, saponins, and honey are superior to placebo and carbocisteine in reducing daytime and nighttime cough and improving the clinical parameters [7].

One of the most studied herbal remedies is *Pelargonium sidoides* extract (EPs 7630). In five randomized studies in children with acute respiratory infections (rhinitis, tonsillopharyngitis, acute bronchitis), a significant reduction in symptom severity, including nasal congestion and cough, as well as a reduction in tonsillitis severity scores, was observed [5]. In acute bronchitis, after 7 days of therapy, a more significant reduction in the severity of bronchitis was observed in the extract group compared to placebo, indicating a rapid onset of clinical effect. The mechanisms of action include antiviral and antibacterial activity, as well as the modulation of tumor necrosis factor-α and nitric oxide production, confirming the anti-inflammatory potential of the plant [49,50]. In all studies, the drug demonstrated a favorable safety profile and good tolerability [5].

A review by Bertoni et al. [5] reported that *Echinacea purpurea* extract was associated with a reduction in cold symptom duration by an average of 1.7 days and a 4–6% decrease in antibiotic prescriptions, based on the pooled data from several studies.

Another promising area is the use of cannabinoids, about which there is little data in pediatrics. A meta-analysis identified eight studies in this field whose methodology allows them to be relied upon. The diseases studied included epilepsy, behavioral problems, chemotherapy-induced nausea and vomiting (CINV), spasticity, and autism spectrum disorders [48].

**Figure 4:**
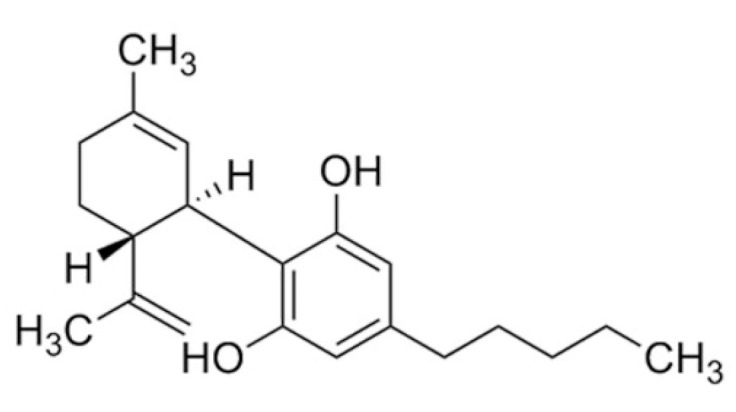
Chemical structure of cannabidiol.

Five studies evaluated the effect of cannabinoids on seizure control, three of which studied pure cannabidiol (CBD, Figure 4) in Dravet syndrome. The analysis showed that CBD is likely to increase the probability of reducing seizure frequency by 50% compared to the baseline. No differences from placebo were found in the overall sample, but in the pure CBD subgroup, a reduction in the number of seizures was noted, while mixed preparations (CBD + D-9-Tetrahydrocannabinol) did not demonstrate this effect. An improvement in the overall clinical picture was also recorded.

In the meta-analysis by Treves et al. [48], two randomized controlled trials showed that nabilone reduces the incidence of chemotherapy-induced vomiting compared to standard antiemetic therapy, although the quality of the studies was low.

A safety analysis of 513 patients indicates a possible increased risk of serious adverse events with the use of medical cannabinoids, although the data are insufficient for reliable conclusions. CBD is associated with decreased appetite and increased gastrointestinal symptoms in children, especially at high doses. Combination preparations did not show such effects. Most studies also reported a higher incidence of psychiatric side effects in the cannabinoid groups. The authors concluded that the use of cannabinoids requires strict control and monitoring of the condition of pediatric patients [48].

From a regulatory perspective, cannabidiol has received FDA approval for two rare, severe pediatric epilepsies—Dravet syndrome and Lennox–Gastaut syndrome—in children aged two years and older, based on several high-quality RCTs. However, no other cannabinoid formulations are currently approved for pediatric indications outside epilepsy, and their use remains off-label or investigational. Importantly, the meta-analysis did not evaluate long-term neurodevelopmental outcomes, but existing evidence from animal and observational studies suggests that exogenous cannabinoid exposure during critical windows of brain maturation may affect memory, learning, and behavior. Therefore, clinicians prescribing cannabinoids to children should implement regular monitoring of growth parameters (especially appetite and weight), gastrointestinal symptoms, neuropsychiatric changes, and liver function tests. Long-term prospective registries are urgently needed to define the safety profile of chronic pediatric cannabinoid use beyond 12–14 weeks [48].

Thus, the results of clinical trials indicate the existence of an evidence base for a number of herbal remedies, provided that age restrictions and standard dosages are observed.

However, there are still gaps in evidence supporting the use of herbal products for the treating of diseases in children. Research is virtually nonexistent on urinary tract, skin, and musculoskeletal infections, as well as fungal infections. The heterogeneity of age groups in the analyzed studies limits the generalizability of the results [5].

## 7. Conclusions

Plant-based biologically active compounds occupy a prominent place in pediatric practice, reflecting both the sustained demand from parents for “natural” treatment methods and the accumulation of experimental and clinical data on their effectiveness. For a number of conditions, primarily acute respiratory infections and functional disorders, convincing results have been obtained from randomized studies confirming the symptomatic benefits of standardized herbal preparations with a satisfactory safety profile. At the same time, interest in their use in neurology, dermatology, and even as adjuvants in cancer therapy is growing.

However, the current evidence base remains heterogeneous, and support for herbal therapies in pediatrics is more limited than often assumed. For most natural compounds and pediatric conditions—beyond a few acute respiratory infections—well-designed randomized controlled trials are lacking or underpowered. Major evidence gaps persist, including long-term safety, age-specific dosing, and drug–herb interactions. Standardization is a critical unresolved issue: variations in plant species, extraction methods, and contaminants (e.g., pyrrolizidine alkaloids) undermine both efficacy and safety. Plant variability poses a major challenge for the pharmaceutical industry in developing standardized, industrially manufactured herbal medicinal products with consistent quality and reproducible clinical effects. Young children are especially vulnerable to differences in composition, yet most herbal products have never been rigorously tested below six years of age. Therefore, while a few standardized products may offer symptomatic benefits in selected acute settings, routine use cannot be recommended without substantial expansion of pediatric-specific trials, strict quality control, and ongoing safety monitoring. A balanced risk–benefit assessment must guide clinical decisions.

## Figures and Tables

**Table 1:** Studies on the simultaneous use of herbal components and drugs.

Herbal Formula	Disease/Condition	Study Design	Concomitant Drugs	Effectiveness	Safety/Interactions	Reference
Curcumin	End-stage chronic kidney disease in children (10–18 years) on regular haemodialysis	Randomized, double-blind, placebo-controlled clinical trial (n = 28, 3 months)	Anticoagulants	No statistically significant difference between groups	All coagulation parameters remained within normal ranges and comparable to placebo	[42]
CUF2 (extract of *Astragalus mongholicus*, *Cordyceps sinensis*, *Radix stemonae*, *Bulbus frutillariae cirrhosae*, *Radix scutellariae*)	Persistent bronchial asthma in children (7–15 years)	Randomized, double-blind, placebo-controlled trial (n = 85, 6 months)	Inhaled corticosteroids	No statistically significant difference between groups	No clinically significant drug interactions or exacerbation of steroid-induced side effects	[43]
Chinese herbal medicines	Epilepsy	Systematic review including 4 studies (n = 246) comparing herbal therapy + drug vs. drug alone	Valproate, carbamazepine, oxcarbazepine	Significantly higher overall efficacy of combined therapy compared to drug monotherapy	Lower incidence of adverse events in combination group, no serious adverse events with herbal therapy alone	[44]
Chinese herbal medicines	Haematologic and solid malignancies	Systematic review of 49 randomized controlled trials	Standard chemotherapy	Significantly increased complete and partial remission rates, improved immune parameters, reduced myelosuppression	Significant reduction in infections, nausea/vomiting, and ECG rhythm abnormalities, no reported increase in adverse events	[45]
*Hypericum perforatum* grapefruit juice *Camellia sinensis* extracts curcumin *Panax ginseng* *Allium sativum* *Zingiber officinale*	Cancer patients receiving chemotherapy/targeted therapy/immunotherapy	Systematic review of clinical and preclinical studies	Chemotherapy, targeted drugs, immunotherapy (nilotinib, sirolimus, bortezomib, 5-fluorouracil, imatinib)	Hypericum perforatum—absolutely contraindicated, grapefruit juice increases nilotinib/sirolimus bioavailability, high-dose green tea inactivates bortezomib, garlic enhances antiplatelet effect	High-risk category: *Hypericum perforatum*, grapefruit juice, *Camellia sinensis* extracts Moderate risk: *Panax ginseng*, curcumin Low risk: *Zingiber officinale*	[46]
Curcumin	Vincristine-induced neuropathy in children (5–15 years) with newly diagnosed acute lymphoblastic leukemia	Randomized, double-blind, placebo-controlled trial (n = 141, 3 months)	Chemotherapy with vincristine	Significantly lower incidence of neuropathy, fewer motor nerve disturbances and signs of lower limb myopathy	No serious adverse events, no clinically significant pharmacokinetic interaction	[47]

**Table 2:** Some clinical studies of herbal components in pediatrics.

	Natural Compounds	Study Design	Number of Patients	Therapeutic Effect	Safety Aspects	Reference
1	Honey	RCT with 4 parallel groups: honey, dextromethorphan, diphenhydramine, control (maintenance therapy)	139	Honey significantly reduces the frequency and severity of nocturnal cough and improves sleep quality in children compared to dextromethorphan, diphenhydramine and supportive care.	In the honey group, two children reported nervousness/hyperactivity. Honey is contraindicated for children under 1 year of age due to the risk of botulism.	[5]
2	Polysaccharide-resin-honey syrup	RCT with 2 parallel groups: PRH syrup and carbocysteine	150	The syrup is significantly more effective than carbocysteine: it reduces the frequency and severity of night and day coughs, reduces discomfort from coughing, and improves the child’s sleep.	Side effects were mild and transient: abdominal pain, nausea, vomiting (5 children in the PRH group, 6 in the carbocysteine group), rash (1 child in each group), and rowsiness (2 in the carbocysteine group).	[7]
3	EPs 7630 (*Pelargonium sidoides* extract)	Randomized, double-blind, placebo-controlled clinical trial with 2 parallel groups: EPs 7630 and placebo	220	After 7 days, there was a significantly greater reduction in the overall bronchitis symptom score compared to placebo. Improved cough and wheezing, improved sleep and appetite, and return to school/kindergarten.	In the EPs 7630 group, 2 patients (1.8%) reported 3 adverse events (gastrointestinal disorder, infection, clinical laboratory parameter changes).	[5]
4	Echinaforce Junior (*Echinacea purpurea* tablets)	A randomized, open-label, parallel clinical trial with two doses (3 × 1 and 5 × 1 tablet/day)	79	Cold episodes last an average of 7.5 days; the dose of 5 tablets/day reduces the duration by 1.2–1.7 days compared to 3 tablets. By day 10, 85% of episodes are completely resolved. Symptoms (sore throat, cough, runny nose) are reduced.	98.5% of physicians rate the treatment as “good” or “very good.” There were 20 adverse events (gastrointestinal tract, infections—scarlet fever, chickenpox, etc.) in 13 children, all mild.	[5]
5	Cannabidiol	Randomized, double-blind, placebo-controlled clinical trial with 2 parallel groups: CBD and placebo	120	CBD is associated with a 50% reduction in seizure frequency in Dravet syndrome.	Increased risk of decreased appetite (especially at the dose of 20 mg/kg/day); increased incidence of adverse psychiatric events (fatigue, drowsiness, irritability, mood changes).	[48]
6	Cannabidiol	Randomized, double-blind, placebo-controlled clinical trial with 2 parallel groups: CBD and placebo	198	A dose-dependent reduction in seizure frequency was observed. A 50% reduction in seizures was achieved in a higher proportion of patients in the CBD group.	The most common adverse events were decreased appetite (at high doses), diarrhea, vomiting, somnolence, and pyrexia. In the CBD group, 72 cases of mental disorders were reported (133 patients) versus 20 (65 patients) in the placebo group.	[48]

## Data Availability

No new data were created or analyzed in this study. Data sharing is not applicable to this article.
